# Migration-related determinants of health-related quality of life of persons with direct migration background in Germany: a study based on the German Socio-Economic Panel

**DOI:** 10.3389/fpubh.2024.1297862

**Published:** 2024-01-29

**Authors:** Thomas Grochtdreis, Hans-Helmut König, Judith Dams

**Affiliations:** Department of Health Economics and Health Services Research, Hamburg Center for Health Economics, University Medical Center Hamburg-Eppendorf, Hamburg, Germany

**Keywords:** health, quality of life, psychological well-being, social determinants of health, transients and migrants

## Abstract

**Introduction:**

Persons with a direct migration background made up a share of around 17% of the total German population in the year 2020. Not much is known about migration-related determinants of health-related quality of life (HrQoL) of persons with direct migration background. This study aimed to analyze the associations between HrQoL, sociodemographic, and migration-related characteristics of persons with direct migration background in Germany.

**Methods:**

The sample of this study was based on four waves (2014, 2016, 2018, and 2020) of the migration samples (M1 and M2) of the German Socio-Economic Panel (SOEP). The SF-12 was used to measure HrQoL using its mental (MCS) and physical (PCS) component summary scores. Missing information was replaced by multiple imputation by chained equations with predictive mean matching. Associations between HrQoL and sociodemographic and migration-related characteristics were examined using multilevel mixed-effects linear regressions.

**Results:**

The mean MCS and PCS scores of persons with direct migration background

(*n* = 4,124) were 51.81 and 51.57, respectively. Being born in Russia was associated statistically significantly with a lower PCS score compared with non-east European and American/Oceanic countries. A longer period since migration to Germany was negatively associated with both MCS and PCS scores (both with *p* < 0.01). A steady relationship before migration was associated with a higher MCS score (+0.69, *p* = 0.017). Not feeling German and experiencing disadvantages due to origin were negatively associated with the MCS (both with *p* < 0.001). The oral ability in the German language was positively associated with the PCS score (*p* < 0.05).

**Conclusion:**

The time since migration to Germany, and the relationship status before migration may be determinants of HrQoL of persons with a direct migration background. Furthermore, connectedness with Germany, disadvantages due to origin and oral ability in the German language, representative of integration in Germany, can be potential determinants of HrQoL. Thus, integration of persons with migration background is crucial for their mental and physical HrQoL.

## 1 Introduction

Persons with a direct migration background, i.e., persons with their own migration experience who were born without German citizenship (1), made up a share of around 17% of the total German population in the year 2020 (2). The group of persons with a direct migration background is relatively heterogeneous in itself since, for example, the country of birth or the reason for migration and, not least, the years of migration to Germany differ fundamentally. Of all persons with a direct migration background, the majority (37%) were born in a country of the European Union (EU-27), followed by a share of 28% that was born in another European country and 26% that were born in an Asian country (3). The main reason for migration to Germany in 2020 was family and partnership by almost half of all persons with a direct migration background followed by economic reasons with a proportion of 18% and refugee/asylum or international protection with a proportion of 16%. Concerning the year of migration to Germany, the foreigner database of the German Federal Statistical Office indicates a 42% increase in the total number of persons with a direct migration background over the last 15 years (2).

Not only the total number of immigrants increased, but also the composition of migration flows has also changed over time. While the fall of the Iron Curtain was a reason for new migration flows until the 2000s, the enlargement of the EU has led to an increase in the number of people with a direct migration background until today (4). Those recent migration flows due to the enlargement of the EU have increasingly been directed toward taking up education or employment, accompanied by an improvement in the levels of qualification of persons with migration background in general (5). As a result, this has led to an increase in persons with a direct migration background in employment in general, but also to an increase in persons with a direct migration background in the ranks of the unemployed.

The increase in the total number of persons with a direct migration background and the change in the composition of migration flows makes it necessary to re-evaluate the existing knowledge about, among other things, the health-related quality of life (HrQoL) and, in particular, the corresponding migration-related determinants of this group of persons (6, 7). In this regard, it is important to acknowledge that the term HrQoL is defined and operationalized inconsistently throughout the literature (8). A common definition of HrQoL is “how well a person functions in their life and his or her perceived wellbeing in physical, mental, and social domains of health” (9). A popular measure of HrQoL that covers the domains addressed by this definition is the SF-6D, which is derived from the SF-12 (10, 11). In this sense, the physical component summary (PCS) and mental component summary (MCS) scores, which are calculated using subscales of the SF-12, can be seen as perceived wellbeing in the physical and mental domains of health and thus as HrQoL.

Recent studies analyzed the HrQoL of persons with direct and indirect migration background, and native-born Germans (12–15). In three of those studies, a positive association between physical HrQoL and migration background has been found (12, 14, 15), whereas one study found a negative association (13). Concerning mental HrQoL, two studies found no association with migration background (13, 14), whereas one study found a negative association between mental HrQoL and Polish migration background (15). One recent study that reduced the imbalance in sociodemographic characteristics between the migrant and non-migrant samples by use of entropy balancing found a positive association between mental HrQoL and migration background (12).

These studies have in common the lack of inclusion of pre- and post-migration-related characteristics that may be determinants of HrQoL (7). However, this is understandable for the reason that these studies compared persons with and without a migration background. Yet, the study of Nesterko et al. (13) also compared different groups of persons with migration background in terms of their migration-related characteristics. An association was found between age at immigration to Germany and physical HrQoL, but not mental HrQoL. The country of birth was also associated with physical HrQoL. Being born in Turkey was associated with the lowest physical HrQoL, and being born in Western European countries with the highest physical HrQoL. However, no association was found between the years since immigration to Germany, German citizenship, and HrQoL (13). A recent study on post-migration stressors and HrQoL among Syrian refugees in Sweden found negative associations between financial and social stress and HrQoL (16). Furthermore, possible associations between HrQoL, competency stress, and discrimination were identified.

It is crucial to understand the associations between HrQoL, sociodemographic characteristics, and migration-related characteristics in order to identify health inequalities and disparities within the group of persons with a direct migration background (17, 18). In order to provide adequate policy implications for policy makers and public health officials with regard to resource allocation and tailoring of health interventions specifically for the still very heterogeneous target group of persons with a direct migration background with respect to improving HrQoL, it is necessary to identify those determinants that contribute to a better or poorer HrQoL (19). For example, future prevention and health promotion programs can be geared more specifically to the target group of persons with a direct migration background if the migration-related determinants of HrQoL are better understood (20).

However, apart from the previously mentioned evidence, to our knowledge, not much is known about potential migration-related determinants of HrQoL among persons with a direct migration background in Germany. To move toward possible explanations for the causes of the aforementioned differences in mental and physical HrQoL of persons with direct migration background in Germany, it is necessary to consider the HrQoL of persons with migration background separately with the inclusion of not only sociodemographic characteristics but also migration-related characteristics as possible determinants of HrQoL. The aim of this study, therefore, was to analyze the associations between HrQoL, sociodemographic, and migration-related characteristics of persons with direct migration background in Germany.

## 2 Materials and methods

### 2.1 Sample

The migration samples (M1 and M2) of the German Socio-Economic Panel (SOEP), a representative German household panel with about 30,000 participants in almost 15,000 households assessed annually since 1984 by the German Institute for Economic Research (DIW Berlin), have been used to compile the sample of this study. To ensure representativeness, the hitherto underrepresented current generation of people with a migration background was added to the SOEP in the past decade using additional samples. The M1 sample was introduced in 2013 with 2,723 households and the M2 sample was introduced in 2015 with 1,096 households. Both samples consist of people aged 18 and older, mainly with an EU migration background, who arrived in Germany in recent years (21). The persons of the migration samples had access to questionnaires in German, English, Russian, Turkish, Polish, and Romanian language (22). Furthermore, an interpreter was optionally available for the interviews.

By 2022, eight consecutive waves of the M1 sample and six waves of the M2 sample were available (waves 30 to 37). As data on the SF-12v2 was available only for the waves of the even years, the sample had to be restricted to waves 31, 33, 35, and 37 (years 2014, 2016, 2018, and 2020). The persons of the four waves (n = 12,673; 29,736 observations) were used to generate a sample of only persons with direct migration background (n = 7,286; 17,039 observations). Thereby, the information on migration background was derived from information on country of birth, citizenship, and parental information using a predefined variable of the SOEP. The sample of persons with direct migration background was further restricted to those without missing information on HrQoL (*n* = 4,124 persons; 9,416 observations).

Data collection took place under strict adherence to the regulations of the EU General Data Protection Regulation (EU-GDPR), the German Federal Data Protection Act (BDSG) as well as all other data protection regulations (23). Thereby, the anonymity of the data was ensured by processing, archiving, and distributing survey data exclusively in anonymous form. Thus, persons are in no way identifiable and personal data is not passed by the DIW Berlin.

### 2.2 Measures

In the SOEP, a modified version of the SF-12v2 was used to measure HrQoL. The SF-12 represents HrQoL on 12 items and the 8 subscales ‘physical functioning’, ‘physical role limitations’, ‘bodily pain’, ‘general health’, ‘vitality’, ‘social functioning’, ‘emotional role limitations’ and ‘mental health’ (24, 25). A modification has been made in the SOEP, in that the question about ‘work interference due to pain’ has been replaced with a question about ‘severe physical pain’ from the SF-36. Besides, the layout, form, and order of the questions of the SF-12 questionnaire were adapted in the SOEP (26, 27).

The subscales ‘physical functioning’, ‘physical role limitations’, ‘social functioning’, and ‘bodily pain’ were used to calculate a PCS score. A MCS score was calculated using the subscales ‘social functioning’, ‘emotional role limitations’, and ‘mental health’. For the Z-transformation of the subscales, a norm-based scoring using mean values and standard deviations of a German normative sample was used (26, 27). As a result, physical and mental HrQoL are represented on the respective summary score on scales ranging between 0 (worst HrQoL) and 100 (best HrQoL).

Furthermore, an SF-6D index score was derived from the SF-12v2 dimensions ‘physical functioning’, ‘role limitations’, ‘social functioning’, ‘pain’, ‘mental health’, and ‘vitality’ using preferences from the general population (10, 11). Theoretically, 7,500 different health states can be defined with those SF-12v2 dimensions that have three to five ordinal levels. For each of the possible health states, index scores anchored at 0 (death) and 1 (full health) were estimated using preference weights derived from the United Kingdom general population (10, 11).

The following sociodemographic characteristics were derived from the SOEP: Sex (female, male), age (18–24, 25–34, 35–44, 45–54, 55–64, and ≥ 65), marital status (married/in partnership, never married/single, widowed, separated/divorced), school-leaving qualification (secondary general school, secondary school, academic secondary school, and no school-leaving qualification), employment (employed, unemployed), citizenship (German, other than German), country of birth and religious affiliation. Country of birth was categorized into Russia, Romania, Kazakhstan, Turkey, other East European countries, other European countries, African countries, other Asian countries, and American/Oceanic countries based on the geographic regions of the Standard Country or Area Codes for Statistical Use (M49) of the United Nations Statistics Division (28). Religious affiliation was categorized into Christian, Muslim, other faith, and non-denominational.

The following migration-related characteristics were derived from the SOEP: Years since the migration to Germany, the main reason for migration (family/partnership reasons, economic reasons, and political reasons), connectedness with the country of origin (And how strongly do you feel connected with your country of origin? Very strong to not at all), feeling German (To what extent do you feel German? Entirely to not at all), steady relationship before migration (Were you in a serious relationship before moving to Germany? Yes and no), and disadvantages due to origin (How often have you personally experienced being disadvantaged in Germany because of your origin? Often, rarely, never). Furthermore, German language skills, in the sense of oral, written, and reading ability, were measured on a scale ranging from 1 (very good) to 5 (not at all).

All interview materials were translated from German into the five languages English, Turkish, Russian, Romanian, and Polish (22). Furthermore, the interviewers were given the opportunity to take an interpreter with them to the interviews.

### 2.3 Statistical analysis

Missing information on the 21 variables used varied between 0.00 and 10.51%, and 3,142 of all 197,739 records (1.59%) were incomplete. Multiple imputation by chained equations with predictive mean matching and a total number of m = 20 imputations was used to improve accuracy and statistical power. The MCS, PCS, SF-6D index, and all sociodemographic and migration-related characteristics analyzed were included in the imputation model.

Descriptive statistics of sociodemographic and migration-related characteristics were calculated. PCS, MCS, and SF-6D index scores were calculated by sociodemographic and migration-related characteristics. Differences in mean MCS, PCS, and SF-6D index scores by sociodemographic and migration-related characteristics were analyzed using F-tests. The descriptive statistics of sociodemographic and migration-related characteristics were calculated based on cross-sectional data, using persons’ data at first occurrence in the selected analytical sample.

Associations between PCS, MCS, and SF-6D index scores and sociodemographic and migration-related characteristics were examined using multilevel mixed-effects linear regressions with the personal identifier as a random effect and cluster robust standard errors. The sociodemographic characteristics gender, age, employment, school-leaving qualification, country of birth, religious affiliation as well as the migration-related characteristics time since the migration to Germany, German citizenship, main reason for migration, connectedness with the country of birth, feeling German, a steady relationship before migration, and disadvantages due to origin were added to the regression models as covariates. Furthermore, only oral ability in the German language was added to the regression models as a covariate, as the variance of oral, written, and reading ability was inflated in the models due to multicollinearity. As a further control variable, the survey year was added to the regression models to take into account for the four waves of the SOEP.

As a sensitivity analysis, data was restricted to the years 2014 to 2018, as the COVID-19 pandemic, which was declared in March 2020 by the WHO, may have had an impact on the HrQoL of persons with direct migration background.

All analyses were performed using Stata/MP 17.0 (StataCorp, College Station, Texas, USA). All statistics were two-sided with a significance level of *p* < 0.05.

## 3 Results

### 3.1 Sociodemographic characteristics

The mean age of the total sample (n = 4,124) was 39 years and 51% of the sample was female. The majority of the sample was married or in a partnership (61%), employed (65%), and affiliated with a Christian religion (53%), yet only 34% had German citizenship (Table 1). About 36% had a secondary general school-leaving qualification, 26% had a secondary school-leaving qualification, and 30% had an academic secondary school-leaving qualification. About 12% of the sample was born in Russia and Turkey, respectively, and about 9% each was born in Romania and Kazakhstan. The other persons in the sample were born in another (Eastern) European country (22%), in another Asian country (21%), in an African country (4%), or an American/Oceanic country (2%). Sociodemographic characteristics of the sample by survey year are shown in Supplementary Table S1 in the Supplementary material.

**Table 1:** Sociodemographic characteristics of the sample (years 2014, 2016, 2018, 2020; *n* = 4,124).

Variables	*N* (%) / Mean (SE)
Sex, female	2,087 (50.61)
Age, mean	38.75 (0.19)
Age categorized	
18–24	518 (12.56)
25–34	1,103 (26.75)
35–44	1,267 (30.72)
45–54	776 (18.82)
55–64	343 (8.32)
≥ 65	117 (2.84)
Marital status	
Married/in partnership	2,518 (61.05)
Never married/single	986 (23.92)
Widowed	266 (6.44)
Separated/divorced	354 (8.59)
School-leaving qualification^a^	
Secondary general school	1,478 (35.85)
Secondary school	1,087 (26.37)
Academic secondary school	1,219 (29.57)
No school-leaving qualification	151 (3.66)
Employment, employed	2,687 (65.16)
Citizenship, German	1,402 (34.00)
Country of birth	
Russia	484 (11.74)
Romania	367 (8.90)
Kazakhstan	362 (8.78)
Turkey	484 (11.74)
Other East European country^b^	406 (9.84)
Other European country^c^	897 (21.75)
African country	152 (3.69)
Other Asian country^d^	874 (21.19)
American/Oceanic country	98 (2.38)
Religious affiliation	
Christian	2,181 (52.90)
Muslim	802 (19.45)
Other faith	186 (4.51)
Non-denominational	955 (23.15)
Health-related quality of life	
MCS, mean	51.81 (0.14)
PCS, mean	51.57 (0.15)
SF-6D index, mean	0.77 (0.00)

SE: Standard error; MCS: mental component summary; PCS: physical component summary.

a ‘Other school-leaving qualification’ is not shown.

b Without Russia, Turkey, and Romania.

c Without East Europe.

d Without Kazakhstan.

### 3.2 Migration-related characteristics

The mean number of years since migration to Germany was 12.59. The majority of persons migrated because of their family or partner (49%), whereas about 32 and 12% migrated for economic and political reasons, respectively (Table 2). Just over half of all persons in the sample stated that they were in a steady relationship before migration (53%). Of all persons in the sample, 48% had a strong or very strong connectedness with their country of birth, whereas 20% had hardly any or no connection at all. The remaining 31% of all persons stated that they were connected in some respects with their country of birth. About 40% of all persons in the sample felt entirely or predominantly German. However, the majority stated that they felt German only in some respects (37%), whereas just 23% stated that they felt hardly or not at all German. Of all persons in the sample, 10% stated that they often experienced disadvantages due to their origin, and 33% stated that they rarely experienced disadvantages. The mean oral, written, and reading ability in the German language was overall good. Migration-related characteristics of the sample by survey year are shown in Supplementary Table S2 in the Supplementary material.

**Table 2:** Migration-related characteristics of the sample (years 2014, 2016, 2018, 2020; *n* = 4,124).

**Variables**	***N* (%) / Mean (SE)**
Years since migration to Germany, mean	12.59 (0.13)
Main reason for migration^a^	
Family/partnership reasons	2,040 (49.48)
Economic reasons	1,308 (31.72)
Political reasons	475 (11.52)
Steady relationship before migration, yes	2,201 (53.37)
Connectedness with country of birth	
Very strong	766 (18.57)
Strong	1,234 (29.92)
In some respects	1,293 (31.34)
Hardly	560 (13.58)
Not at all	272 (6.60)
Feeling German	
Entirely	607 (14.72)
Predominantly	1,039 (25.19)
In some respects	1,533 (37.17)
Hardly	598 (14.49)
Not at all	347 (8.42)
Disadvantages due to origin	
Often	411 (9.96)
Rarely	1,379 (33.43)
Never	2,335 (56.61)
German language skills^b^	
Oral ability, mean	2.22 (0.02)
Written ability, mean	2.44 (0.02)
Reading ability, mean	2.20 (0.02)

SE: Standard error.

a ‘Other main reason for migration’ is not shown.

b Range: 1 (very good) to 5 (not at all).

### 3.3 Descriptive statistics

The mean PCS and MCS, and SF-6D index score of the total sample were 51.81, 51.57, and 0.77, respectively (Table 1). The mean PCS and MCS, and SF-6D index scores by sociodemographic and migration-related characteristics are shown in Supplementary Tables S3, S4 in the online Supplementary material.

### 3.4 Regression analysis

The multilevel mixed-effects linear regressions of MCS and PCS scores in persons with direct migration background showed no associations with the sociodemographic characteristics gender, marital status, and school-leaving qualification (Table 3). A higher age, however, was statistically significantly associated with a lower PCS score (−0.30, *p* < 0.001), and being employed was statistically significantly associated with both, a higher MCS (+1.95) and PCS (+2.80) score (both with *p* < 0.001). The country of birth was not associated with the MCS score. Being born in European countries without East European countries (+1.02, *p* = 0.029), and American/Oceanian countries (+3.07, *p* < 0.001), however, was statistically significantly associated with a higher PCS score compared with being born in Russia. Being affiliated with a Christian religion was associated with a higher MCS score compared with being non-denominational (+0.83, *p* = 0.002).

**Table 3:** Multilevel mixed-effects linear regressions of MCS and PCS scores and selected sociodemographic and migration-related characteristics with cluster robust standard errors (years 2014, 2016, 2018, 2020; *n* = 4,124; 9,419 observations).

Variable	Model 1 (dependent variable MCS)			Model 2 (dependent variable PCS)		
	Coeff.	95% CI	*p value*	Coeff.	95% CI	*p value*
Gender (Ref. male)						
Female	−0.02	−0.35; 0.32	0.929	0.02	−0.30; 0.34	0.918
Age, years	0.02	−0.01; 0.04	0.138	*−* *0.30*	*−0.32*; *−0.27*	*< 0.001*
Marital status (Ref. married/in partnership)						
Never married/single	0.02	−0.50; 0.53	0.954	0.27	−0.22; 0.77	0.277
Widowed	−0.02	−0.83; 0.78	0.954	−0.59	−1.35; 0.17	0.129
Separated/divorced	−0.09	−0.84; 0.66	0.813	−0.15	−0.85; 0.54	0.662
Employment (Ref. unemployed)						
Employed	*1.95*	*1.48; 2.42*	*< 0.001*	*2.80*	*2.34; 3.26*	*< 0.001*
School-leaving qualification (Ref. secondary general school)^a^						
Secondary school	−0.25	−0.76; 0.26	0.333	−0.29	−0.76; 0.18	0.224
Academic secondary school	0.04	−0.45; 0.54	0.870	0.14	−0.34; 0.62	0.554
No school-leaving qualification	−0.79	−1.95; 0.37	0.182	−0.38	−1.56; 0.81	0.532
Country of birth (Ref. Russia)						
Romania	0.82	−0.17; 1.80	0.105	0.56	−0.45; 1.58	0.278
Kazakhstan	0.67	−0.28; 1.62	0.166	0.75	−0.25; 1.75	0.143
Turkey	−0.49	−1.43; 0.45	0.307	0.02	−0.97; 1.01	0.969
Other East Europe^b^	0.60	−0.42; 1.62	0.246	0.48	−0.54; 1.50	0.355
Other Europe^c^	−0.32	−1.25; 0.61	0.497	*1.02*	*0.10; 1.94*	*0.029*
Other Asia^d^	−0.21	−1.20; 0.77	0.670	−0.57	−1.58; 0.44	0.267
Africa	0.71	−0.58; 2.00	0.282	−0.46	−1.87; 0.59	0.520
America/Oceania	−0.71	−2.48; 1.05	0.429	*3.07*	*1.58; 4.57*	*< 0.001*
Religious affiliation (Ref. Christian)						
Muslim	−0.05	−0.84; 0.74	0.904	*−0.92*	*−1.69; −0.14*	*0.020*
Other faith	0.85	−0.38; 2.07	0.175	0.54	−0.77; 1.86	0.419
Non-denominational	*−0.83*	*−1.35; −0.31*	*0.002*	0.37	−0.14; 0.88	0.158
Time since migration to Germany, years	*−0.11*	*−0.15; −0.07*	*< 0.001*	*−0.06*	*−0.10; −0.02*	*0.002*
German citizenship (Ref. yes)						
No	0.32	−0.25; 0.88	0.272	0.18	−0.39; 0.75	0.536
Main reason for migration (Ref. family/partnership reasons)^e^						
Economic reasons	−0.14	−0.70; 0.42	0.628	0.14	−0.41; 0.70	0.611
Political reasons	−0.55	−1.41; 0.30	0.205	−0.11	−1.01; 0.79	0.814
Steady relationship before migration (Ref. yes)						
No	*−0.69*	*−1.26; −0.12*	*0.017*	−0.40	−1.00; 0.20	0.192
Connectedness with country of birth (Ref. very strong)						
Strong	0.33	−0.25; 0.91	0.264	0.38	−0.17; 0.92	0.173
In some respects	−0.02	−0.65; 0.60	0.947	−0.14	−0.72; 0.44	0.640
Hardly	0.22	−0.54; 0.97	0.576	0.18	−0.53; 0.88	0.626
Not at all	0.69	−0.27; 1.65	0.160	−0.39	−1.31; 0.54	0.416
Feeling German (Ref. entirely)						
Predominantly	*−1.21*	*−1.82; −0.61*	*< 0.001*	0.02	−0.52; 0.56	0.937
In some respects	*−1.71*	*−2.33; −1.08*	*< 0.001*	−0.29	−0.86; 0.29	0.327
Hardly	*−1.70*	*−2.47; −0.93*	*< 0.001*	*−0.94*	*−1.67; −0.20*	*0.013*
Not at all	*−1.80*	*−2.77; −0.82*	*< 0.001*	−0.31	−1.20; 0.58	0.495
Disadvantages due to origin (Ref. often)						
Rarely	*1.72*	*0.85; 2.59*	*< 0.001*	0.21	−0.58; 0.99	0.606
Never	*3.01*	*2.15; 3.88*	*< 0.001*	*1.11*	*0.35; 1.88*	*0.004*
Oral ability in the German language (Ref. very good)						
Good	0.01	−0.52; 0.54	0.964	*−0.60*	*−1.08; −0.13*	*0.013*
Not bad	−0.22	−0.88; 0.44	0.519	*−1.11*	*−1.72; −0.51*	*< 0.001*
Fairly bad	−0.65	−1.58; 0.29	0.175	*−1.80*	*−2.69; −0.91*	*< 0.001*
Not at all	−1.63	−4.01; 0.75	0.180	−1.88	−3.96; 0.20	0.076
Survey year (Ref. 2014)						
2016	*0.63*	*0.21; 1.06*	*0.004*	−0.17	−0.55; 0.21	0.384
2018	0.12	−0.37; 0.61	0.628	0.04	−0.41; 0.48	0.876
2020	*−1.40*	*−1.96; −0.83*	*< 0.001*	−0.02	−0.52; 0.48	0.927
Constant	*50.49*	*48.80; 52.19*	*< 0.001*	*62.03*	*60.39; 63.68*	*< 0.001*

CI: confidence interval.

a ‘Other school-leaving qualification’ is not shown.

b Without Russia, Turkey, and Romania.

c Without East Europe.

d Without Kazakhstan.

e ‘Other main reason for migration’ is not shown.

No associations with the migration-related characteristics ‘German citizenship’, ‘connectedness with country of birth’, and the ‘main reason for migration’ could be shown in the multilevel mixed-effects linear regressions of MCS and PCS scores. However, a longer time since migration to Germany was negatively associated with both MCS (*p* < 0.001) and PCS scores (*p* = 0.002). A steady relationship before migration was associated with a statistically significantly higher MCS score (+0.69, *p* = 0.017).

The more persons with a direct migration background felt German, the higher their MCS was. For example, the MCS score of persons who felt entirely German was statistically significantly higher by 1.80 with *p* < 0.001 compared with persons who felt not at all German. Furthermore, persons who rarely (+1.72) or never (+3.01) experienced disadvantages due to their origin had statistically significantly higher MCS scores compared with those who often experienced disadvantages due to their origin (both with *p* < 0.001). Persons who never experienced disadvantages due to their origin also had statistically significantly higher PCS scores compared with those who often experienced disadvantages due to their origin (+1.11, *p* = 0.004). The oral ability in the German language was also positively associated with the PCS score. For example, the PCS score of persons with a very good oral ability in the German language was statistically significantly higher by 1.80 compared with persons with a fairly bad oral ability in the German language (*p* < 0.001). Yet, the oral ability in the German language was not associated with the MCS score.

The results of the multilevel mixed-effects linear regression of SF-6D index scores and sociodemographic and migration-related characteristics in persons with direct migration background are shown in Supplementary Table S5 in the Supplementary material. A longer time since migration to Germany (*p* < 0.001) and feeling less German (all with *p* < 0.050) were negatively associated with the SF-6D index score, a steady relationship before migration (*p* = 0.025), and experiencing fewer disadvantages due to origin (all with *p* < 0.050) were positively associated with the SF-6D index score. The sensitivity analysis without the COVID-19 pandemic year 2020 did not alter the results of the multilevel mixed-effects linear regression of the MCS and PCS scores (Supplementary Table S6 in the Supplementary material).

## 4 Discussion

This was the first study to analyze the associations between HrQoL, sociodemographic and, in particular, migration-related characteristics of persons with a direct migration background in Germany. The incorporation of migration-related characteristics into the multilevel mixed-effects linear regression analyses was an important step toward understanding migration-related determinants of HrQoL and thus toward identifying health inequalities and disparities within this group of persons. It is now crucial to be in a better position to know what contributes to a better or poorer HrQoL in the very heterogeneous group of persons with a direct migration background presented so far, in order to be able to take adequate policy measures and, last but not least, to improve public health.

It can therefore be stated primarily that mental and physical HrQoL was not associated with gender, marital status, and school-leaving qualification, whereas a higher age was negatively associated with physical HrQoL and employment was positively associated with both mental and physical HrQoL. It has already been assumed that reduced HrQoL, especially among persons with migration background, may be negatively associated with education, employment, and social activities (29). We can at least confirm that employment can be a determinant of HrQoL in persons with direct migration background.

Furthermore, being affiliated with a Christian religion was associated with a higher mental HrQoL compared with being non-denominational. Religiosity is generally positively associated with mental well-being and quality of life, and specifically with mental HrQoL among Eritrean refugees in Norway, with no differences found between the religious affiliations (30–32). Being affiliated with a religion may be therefore a protective factor against mental distress as well as a supportive factor for good mental HrQoL among persons with a migration background (30, 33).

Being born in Russia was negatively associated with physical HrQoL compared to other non-Eastern European countries, and America/Oceania. The highly positive association between being born in America/Oceania might be related to the ‘healthy migrant effect’ of Western labor market-oriented migrants immigrating voluntarily to European countries (34, 35). Persons with Russian migration background who immigrated after the fall of the Iron Curtain could generally have a poorer HrQoL as well as a lower life expectancy. According to the OECD Better Life Index, persons in the Russian Federation are below average in terms of health and life satisfaction (36). Life expectancy at birth in the Russian Federation was 73 years, lower than the OECD average of 81 years. The overall normative value of the EQ-5D-3L index in the five largest European economies was 0.92, while the corresponding index value in the Russian Federation was 0.84, indicating a lower HrQoL (37, 38). In addition, differences in mental and physical HrQoL among persons of different origins could also be due to different interpretations of the meaning of and responses to the items of the SF-12 (39, 40).

With respect to being born in Russia, the study by Nesterko et al. (13) found a positive association with mental HrQoL, but no association with physical HrQoL, while being born in Turkey, Poland and other Eastern European countries was negatively associated with physical HrQoL. In the current study, for being born in Turkey and in other Eastern European countries, no association with HrQoL has been found. While the argumentation of the above-mentioned study was based on the adverse employment situations because of labor migration up to the 1970s, this may not be true for the more recent migration flows up to the 2000s or until today (13, 41).

With respect to migration-related characteristics, it has been identified that mental and physical HrQoL was negatively associated with time since migration to Germany. In earlier studies, a younger age at immigration to Germany was negatively associated with physical HrQoL as well as negatively associated with the mental health of corresponding children (14, 42). Furthermore, having a steady relationship before migration was found to be positively associated with mental HrQoL in the current study. According to the life course framework of migration and health, individual exposures in the country of origin, such as having a steady relationship before migration, may have an influence on the health situation and thus probably also on the HrQoL of persons with direct migration background (43).

The reason for migration was neither associated with mental, nor with physical HrQoL in the current study. It has already be shown that the reason for migration was negatively associated with mental health (44, 45), i.e., political reasons were associated with higher prevalence rates of depression and anxiety than economic reasons, which could not be confirmed by us in connection with mental HrQoL.

The mental HrQoL was positively associated with feeling German. Yet, if persons with a direct migration background often experienced disadvantages due to their origin, mental HrQoL was lower. Consistent with this, HrQoL was negatively associated with a higher social stress and high discrimination among Syrian refugees in Sweden (16). The integration of persons with migration background, where feeling as a person of the country of immigration or knowledge of the national language can be seen as proxies, is possibly associated with health and quality of life (29). In the current study, the physical HrQoL was positively associated with the oral ability in the German language. As oral ability might be an informal facilitator to accessing health care, it might thereby also have an influence on HrQoL (29, 46, 47). A worse ability in the German language of parents with direct migration background was directly negatively associated with the mental health of their children (42).

### 4.1 Comparability and generalizability

Because norm-based physical and mental HrQoL, and HrQoL using preference-weighted data have rarely been used to assess the associations between HrQoL and migration-related characteristics in migrant samples, comparability is limited to one study by Sengoelge et al. (16). In the current study, the preference-based HrQoL was associated with the migration-related characteristics ‘time since migration to Germany’, ‘feeling German’, ‘steady relationship before migration’, and ‘experiencing disadvantages due to origin’. In the study by Sengoelge et al. (16), preference-based HrQoL was also negatively associated with discrimination and post-migration stressors, but not with the year of immigration. Yet, the year of immigration was only included in the model as a categorical variable (before 2012, 2012, and 2013), while the time since migration to Germany is a metric variable in the model of the current study.

The mental (51.8 vs. 50.9) and physical HrQoL (51.6 vs. 51.5) of the total sample of persons with migration background was slightly higher and merely identical, respectively, compared with the HrQoL of another representative migrant sample in Germany (12). Compared with the HrQoL of the German normative sample of the SF-12 (50.0), both the mental and physical HrQoL were higher in the current sample of persons with direct migration background (26, 27). The mean age of the sample was 39 years, which is comparable to the mean age of persons with a direct migration background in Germany who have immigrated over the last 30 years (mean age 37 years) according to the foreigners database of the German Federal Statistical Office (3).

As the data of the SOEP has the claim to representativeness of German households and as the additional migrant samples were integrated in the SOEP to better represent the current generation of people with a migration background, at least an indication of representativeness of persons with direct migration background is given. In these migrant samples, however, certain groups of persons, e.g., from the new Eastern EU countries and the southern EU member states, were deliberately overrepresented (22). Thereby, generalizability of the results of the present study with regard to the association between HrQoL, sociodemographic, and migration-related characteristics of persons with a direct migration background in Germany is thus possibly given with restrictions with regard to the overrepresentation of certain groups of persons.

### 4.2 Policy implications

Persons with a direct migration background are exposed to various factors that contribute to a poorer HrQoL. Besides the migration-related factors that have been identified in the current study, also environmental and social conditions in the country of origin as well as in Germany, such as working conditions and socio-economic status, play a certain role (43, 48–50). In addition to improving environmental and social conditions, equal access to health care should also be strived for, i.e., the provision of low-threshold health care services, the minimization of cultural stereotypes in the health care system, target group-specific health communication, and the reduction of language barriers (48, 51–53). Prevention and health promotion needs to target the most vulnerable groups of persons with a direct migration background with tailored and low-threshold programs (19). In particular, mental HrQoL needs to be addressed through accessible and culturally sensitive mental health support programs. Destigmatization of mental health services and outreach efforts toward persons with a direct migration background are one key to a more successful mental health support (54).

There is also an urgent need for persons with a direct migration background to be better connected to Germany and for disadvantages due to their origin to be prevented by the government and society. Finally, yet importantly, it is essential to keep the integration of persons with migration background in general by means of, e.g., the provision of German language learning and the creation of good conditions on the labor market, in the focus of policies. Overall, both health care and social policies need to take a holistic and integrative approach to acknowledge the heterogeneity of the group of persons with a direct migration background.

### 4.3 Strengths and limitations

One major strength of this study was the use of the recently integrated additional migrant samples from a German household panel, which ensures the representation of persons with migration background proportionally to the German population. By the availability of questionnaires in multiple languages and an optional interpreter, bias was reduced with respect to selection of, e.g., higher educated persons or persons with better German language skills. Secondly, to our knowledge, this was the first study systematically including several migration-related characteristics as covariates in the regression models examining associations with mental and physical HrQoL of persons with direct migration background in Germany. This made it possible to take into account the heterogeneity of the group of persons with a direct migration background.

However, this study has some limitations to be mentioned. Firstly, since no analysis plan was registered before the analyses were conducted, the obligation to undertake analytical steps without prior knowledge of the research results cannot be proven (55). Therefore, the current study must be regarded as exploratory rather than confirmatory. Consequently, the statistical inference tests should be interpreted with caution, as multiple comparisons may have inflated the Type I error. Secondly, despite the availability of data from observations of four waves of the SOEP, it was not possible to draw conclusions about the causal relationship of sociodemographic determinants, migration-related determinants, and HrQoL. The determinants used were largely constant over time, so that only associations could be analyzed. Thirdly, the SF-6D index scores were estimated using preference weights derived from the United Kingdom general population (10, 11). As no preference data was available from the German population or any other population group relevant to persons with a direct migration background in Germany, this can be considered the best available option. Furthermore, the main analysis focused on physical and mental HrQoL, presented as PCS and MCS scores, whose subscales were Z-transformed using means and standard deviations of a German normative sample (20, 21). In this way, a potential reduction in validity due to the association of cultural values with self-reported health and HrQoL was overcome (56). Fourthly, the fourth wave of the SOEP that has been used in this study was collected during the first year of the COVID-19 pandemic, so the associations between HrQoL, sociodemographic and migration-related characteristics may have been indirectly moderated by this. Yet, a sensitivity analysis without the COVID-19 pandemic year 2020 showed no evidence of such a moderation effect.

## 5 Conclusion

In addition to the previously known sociodemographic determinants of HrQoL of persons with a direct migration background, employment, and country of birth, this study also reinforces that the time since migration to Germany and the relationship status before migration may be also determinants of HrQoL. To our knowledge, an association between connectedness with Germany, disadvantages due to their origin, and oral ability in the German language and HrQoL could be demonstrated for the first time. Thus, special attention must be paid to the integration of persons with migration background as potential determinant of both mental and physical HrQoL. Further research is needed to better understand the causal relationship between migration-related determinants and HrQoL, as well as qualitative research on the meaning of these determinants for persons with a direct migration background. Particular attention should also be paid to flight-related determinants of HrQoL among asylum seekers and refugees in Germany.

## Data availability statement

The data analyzed in this study is subject to the following licenses/restrictions: The data can be applied for via the website of the German Institute for Economic Research (DIW), Berlin. It is available free of charge for scientific, non-commercial use. The code used during the current study is available from the corresponding author on reasonable request for all interested researchers. Requests to access these datasets should be directed to t.grochtdreis@uke.de.

## Ethics statement

This study involves human participants but was not approved since the criteria for the need of an ethical statement were not fulfilled (e.g., examination of patients or risk for the respondents). Nevertheless, the German Council of Science and Humanities (Wissenschaftsrat) evaluated and approved the German Socio-Economic Panel (SOEP). Participants gave written informed consent to participate in this study before taking part in accordance with the national legislation and the requirements of the German Institute for Economic Research (DIW).

## Author contributions

TG: Conceptualization, Data curation, Formal analysis, Methodology, Writing – original draft, Writing – review & editing. H-HK: Formal analysis, Supervision, Writing – review & editing. JD: Formal analysis, Supervision, Writing – review & editing.
