# Supplementary material for: Migration-related determinants of health-related quality of life of persons with direct migration background in Germany: a study based on the German Socio-Economic Panel
Source: Front Public Health. 2024 Jan 29;12:1297862. doi: 10.3389/fpubh.2024.1297862 (PMC10859458; doi:10.3389/fpubh.2024.1297862)
Supplement: Supplementary file 1 [file Table_1.DOCX]

**Supplementary Table 1. Sociodemographic characteristics of the sample by survey year (n = 4124; 9419 observations)**

| **Variables** | **2014** | **2016** | **2018** | **2020** | ***p*-value^e^** |
| --- | --- | --- | --- | --- | --- |
|  | **N (%) / Mean (SE)** | | | |  |
| Sex, female | 1383 (50.88) | 1447 (50.49) | 1126 (50.74) | 823 (50.93) | 0.990 |
| Age, mean | 40.19 (0.23) | 40.27 (0.23) | 42.28 (0.27) | 43.93 (0.32) | < 0.001 |
| 18-24 | 298 (10.96) | 253 (8.83) | 161 (7.26) | 95 (5.88) | < 0.001 |
| 25-34 | 617 (22.70) | 758 (26.45) | 497 (22.40) | 302 (18.69) |  |
| 35-44 | 862 (31.71) | 888 (30.98) | 668 (30.10) | 501 (31.00) |  |
| 45-54 | 575 (21.16) | 587 (20.48) | 502 (22.62) | 385 (23.82) |  |
| 55-64 | 279 (10.26) | 274 (9.56) | 272 (12.26) | 204 (12.62) |  |
| ≥ 65 | 87 (3.20) | 106 (3.70) | 119 (5.36) | 129 (7.98) |  |
| Marital status |  |  |  |  |  |
| Married/in partnership | 1680 (61.8) | 1710 (59.65) | 1319 (59.45) | 962 (59.50) | 0.810 |
| Never married/single | 643 (23.64) | 718 (25.04) | 554 (24.95) | 410 (25.37) |  |
| Widowed | 167 (6.14) | 197 (6.88) | 161 (7.24) | 109 (6.72) |  |
| Separated/divorced | 229 (8.43) | 242 (8.44) | 186 (8.36) | 136 (8.41) |  |
| School-leaving qualification^a^ |  |  |  |  |  |
| Secondary general school | 1027 (37.77) | 918 (32.02) | 478 (21.54) | 297 (18.39) | < 0.001 |
| Secondary school | 727 (26.74) | 725 (25.31) | 563 (25.37) | 331 (20.45) |  |
| Academic secondary school | 735 (27.03) | 1032 (36.01) | 1056 (47.57) | 931 (57.61) |  |
| No school-leaving qualification | 105 (3.87) | 84 (2.94) | 53 (2.40) | 25 (1.55) |  |
| Employment, employed | 1809 (66.56) | 1921 (67.03) | 1526 (68.77) | 1082 (66.96) | 0.385 |
| Citizenship, German | 1162 (42.75) | 982 (34.26) | 828 (37.31) | 690 (42.70) | < 0.001 |
| Country of birth |  |  |  |  |  |
| Russia | 404 (14.86) | 342 (11.93) | 263 (11.85) | 206 (12.75) | < 0.001 |
| Romania | 205 (7.54) | 270 (9.42) | 215 (9.69) | 173 (10.71) |  |
| Kazakhstan | 307 (11.30) | 263 (9.18) | 190 (8.56) | 130 (8.04) |  |
| Turkey | 304 (11.18) | 327 (11.41) | 264 (11.90) | 180 (11.14) |  |
| Other East European country^b^ | 182 (6.70) | 296 (10.33) | 240 (10.82) | 184 (11.39) |  |
| Other European country^c^ | 598 (22.00) | 617 (21.53) | 451 (20.32) | 337 (20.85) |  |
| African country | 84 (3.09) | 100 (3.49) | 72 (3.24) | 51 (3.16) |  |
| Other Asian country^d^ | 592 (21.78) | 582 (20.31) | 476 (21.45) | 314 (19.43) |  |
| American/Oceanic country | 42 (1.55) | 69 (2.41) | 48 (2.16) | 41 (2.54) |  |
| Religious affiliation |  |  |  |  |  |
| Christian | 1415 (52.07) | 1550 (54.1) | 1185 (53.38) | 833 (51.52) | < 0.001 |
| Muslim | 546 (20.10) | 528 (18.43) | 432 (19.48) | 251 (15.53) |  |
| Other faith | 120 (4.40) | 123 (4.30) | 104 (4.69) | 54 (3.36) |  |
| Non-denominational | 637 (23.43) | 664 (23.17) | 498 (22.45) | 478 (29.59) |  |
| Health-related quality of life | 1383 (50.88) | 1447 (50.49) | 1126 (50.74) | 823 (50.93) |  |
| MCS, mean | 40.19 (0.23) | 40.27 (0.23) | 42.28 (0.27) | 43.93 (0.32) | < 0.001 |
| PCS, mean | 298 (10.96) | 253 (8.83) | 161 (7.26) | 95 (5.88) | 0.003 |
| SF-6D index, mean | 617 (22.70) | 758 (26.45) | 497 (22.40) | 302 (18.69) | < 0.001 |

SE: Standard error; MCS: mental component summary; PCS: physical component summary

^a^ ‘Other school-leaving qualification’ is not shown

^b^ Without Russia, Turkey, and Romania

^c^ Without East Europe

^d^ Without Kazakhstan

^e^ Comparison of sociodemographic characteristics between survey years analyzed using F-test

**Supplementary Table 2. Migration-related characteristics of the sample by survey year (n = 4124; 9419 observations)**

| **Variables** | **2014** | **2016** | **2018** | **2020** | ***p*-value^c^** |
| --- | --- | --- | --- | --- | --- |
|  | **N (%) / Mean (SE)** | | | |  |
| Years since migration to Germany, mean | 14.92 (0.16) | 13.34 (0.17) | 15.15 (0.19) | 17.19 (0.22) | < 0.001 |
| Main reason for migration^a^ |  |  |  |  |  |
| Family/partnership reasons | 1437 (52.86) | 1362 (47.51) | 1051 (47.38) | 749 (46.34) | < 0.001 |
| Economic reasons | 782 (28.77) | 963 (33.62) | 752 (33.87) | 551 (34.10) |  |
| Political reasons | 295 (10.85) | 326 (11.39) | 255 (11.50) | 188 (11.62) |  |
| Steady relationship before migration, yes | 1331 (48.98) | 1606 (56.04) | 1224 (55.15) | 861 (53.29) |  |
| Connectedness with country of birth |  |  |  |  |  |
| Very strong | 482 (17.73) | 511 (17.84) | 381 (17.18) | 246 (15.25) | 0.1928 |
| Strong | 779 (28.66) | 856 (29.87) | 622 (28.02) | 447 (27.67) |  |
| In some respects | 872 (32.08) | 915 (31.93) | 730 (32.92) | 569 (35.20) |  |
| Hardly | 390 (14.35) | 407 (14.22) | 347 (15.62) | 239 (14.77) |  |
| Not at all | 195 (7.17) | 176 (6.14) | 139 (6.27) | 115 (7.12) |  |
| Feeling German |  |  |  |  |  |
| Entirely | 437 (16.09) | 486 (16.94) | 320 (14.44) | 552 (34.13) | < 0.001 |
| Predominantly | 717 (26.36) | 849 (29.61) | 666 (30.01) | 573 (35.45) |  |
| In some respects | 955 (35.14) | 1016 (35.45) | 786 (35.44) | 382 (23.62) |  |
| Hardly | 382 (14.06) | 347 (12.12) | 269 (12.13) | 103 (6.34) |  |
| Not at all | 227 (8.35) | 168 (5.87) | 177 (7.98) | 7 (0.45) |  |
| Disadvantages due to origin |  |  |  |  |  |
| Often | 319 (11.75) | 175 (6.11) | 127 (5.73) | 83 (5.11) | < 0.001 |
| Rarely | 1021 (37.56) | 747 (26.06) | 655 (29.54) | 466 (28.85) |  |
| Never | 1378 (50.69) | 1944 (67.83) | 1437 (64.74) | 1067 (66.05) |  |
| German language skills^b^ |  |  |  |  |  |
| Oral ability, mean | 2.10 (0.02) | 2.24 (0.02) | 2.18 (0.02) | 2.04 (0.02) | < 0.001 |
| Written ability, mean | 2.34 (0.02) | 2.45 (0.02) | 2.38 (0.02) | 2.24 (0.03) | < 0.001 |
| Reading ability, mean | 2.09 (0.02) | 2.22 (0.02) | 2.15 (0.02) | 2.00 (0.02) | < 0.001 |

SE: Standard error

^a^ ‘Other main reason for migration’ is not shown

^b^ Range: 1 (very good) to 5 (not at all)

^c^ Comparison of sociodemographic characteristics between survey years analyzed using F-test

Supplementary Table 3. Mean MCS, PCS and SF-6D index scores by sociodemographic characteristics (n = 4124)

|  | **N** | **MCS** | **PCS** | **SF-6D index** |
| --- | --- | --- | --- | --- |
|  |  | Mean (SE) | | |
| Gender |  |  |  |  |
| Female | 2087 | 51.80 (0.20) | 51.37 (0.22) | 0.77 (0.00) |
| Male | 2037 | 51.82 (0.21) | 51.77 (0.22) | 0.77 (0.00) |
| Age |  |  |  |  |
| 18-24 | 518 | 52.16 (0.41) | 55.67 (0.40)*** | 0.81 (0.01)*** |
| 25-34 | 1103 | 51.92 (0.28) | 54.90 (0.27) | 0.80 (0.00) |
| 35-44 | 1267 | 51.78 (0.26) | 52.03 (0.26) | 0.77 (0.00) |
| 45-54 | 776 | 51.25 (0.33) | 48.59 (0.33) | 0.74 (0.00) |
| 55-64 | 343 | 52.15 (0.50) | 43.58 (0.49) | 0.71 (0.01) |
| ≥ 65 | 117 | 52.31 (0.86) | 40.31 (0.84) | 0.69 (0.01) |
| Marital status |  |  |  |  |
| Never married/single | 986 | 51.73 (0.32) | 51.89 (0.34) | 0.77 (0.00) |
| Married/in partnership | 2518 | 51.86 (0.19) | 51.54 (0.20) | 0.77 (0.00) |
| Separated/divorced | 354 | 51.72 (0.56) | 51.47 (0.57) | 0.77 (0.01) |
| Widowed | 266 | 51.75 (0.62) | 50.79 (0.63) | 0.76 (0.01) |
| School-leaving qualification^a^ |  |  |  |  |
| Secondary general school | 1478 | 51.94 (0.24) | 51.49 (0.26) | 0.77 (0.00) |
| Secondary school | 1087 | 51.52 (0.29) | 51.32 (0.31) | 0.76 (0.00) |
| Academic secondary school | 1219 | 51.91 (0.27) | 51.99 (0.29) | 0.77 (0.00) |
| No school-leaving qualification | 151 | 51.17 (0.79) | 51.24 (0.84) | 0.76 (0.01) |
| Employment |  |  |  |  |
| Employed | 2687 | 52.49 (0.18)*** | 53.00 (0.19)*** | 0.79 (0.00)*** |
| Unemployed | 1437 | 50.54 (0.24) | 48.89 (0.26) | 0.74 (0.00) |
| Nationality |  |  |  |  |
| German | 1402 | 51.68 (0.25) | 50.77 (0.26)*** | 0.76 (0.00)* |
| Other nationality | 2722 | 51.88 (0.18) | 51.98 (0.19) | 0.77 (0.00) |
| Country of birth |  |  |  |  |
| Russia | 484 | 51.78 (0.42)*** | 49.59 (0.45)*** | 0.75 (0.01)*** |
| Romania | 367 | 54.11 (0.48) | 53.15 (0.51) | 0.81 (0.01) |
| Kazakhstan | 362 | 52.64 (0.49) | 51.49 (0.52) | 0.78 (0.01) |
| Turkey | 484 | 51.52 (0.42) | 52.15 (0.45) | 0.77 (0.01) |
| Other East European country^b^ | 406 | 52.95 (0.46) | 52.70 (0.49) | 0.78 (0.01) |
| Other European country^c^ | 897 | 51.32 (0.31) | 52.29 (0.33) | 0.77 (0.00) |
| Other Asian country^d^ | 874 | 50.69 (0.31) | 50.23 (0.33) | 0.75 (0.00) |
| African country | 152 | 52.10 (0.75) | 51.01 (0.80) | 0.76 (0.01) |
| American/Oceanic country | 98 | 51.13 (0.93) | 54.42 (0.99) | 0.77 (0.01) |
| Religious affiliation |  |  |  |  |
| Christian | 2181 | 52.40 (0.20)*** | 51.75 (0.21)*** | 0.78 (0.00)*** |
| Muslim | 802 | 50.68 (0.33) | 50.13 (0.36) | 0.75 (0.00) |
| Other faith | 186 | 52.06 (0.70) | 51.12 (0.76) | 0.76 (0.01) |
| Non-denominational | 955 | 51.37 (0.30) | 52.46 (0.32) | 0.77 (0.00) |

Comparisons of mean MCS, PCS, and SF-6D index scores by sociodemographic characteristics were analyzed using F tests

MCS: Mental Component Summary; PCS: Physical Component Summary; SE: standard error

^a^ ‘Other school-leaving qualification’ is not shown

^b^ Without Russia, Turkey, and Romania

^c^ Without East Europe

^d^ Without Kazakhstan

* p<0.05, ** p≤0.01, *** p≤0.001

**Supplementary Table 4. Mean MCS, PCS and SF-6D index scores by migration-related characteristics (n = 4124)**

|  | **N** | **MCS** | **PCS** | **SF-6D index** |
| --- | --- | --- | --- | --- |
|  |  | Mean (SE) | | |
| Years since migration to Germany |  |  |  |  |
| 0-4 | 687 | 53.58 (0.35)*** | 53.92 (0.37)*** | 0.81 (0.01)*** |
| 5-9 | 1040 | 52.15 (0.29) | 52.88 (0.30) | 0.78 (0.00) |
| 10-14 | 856 | 52.39 (0.32) | 51.18 (0.33) | 0.77 (0.00) |
| 15-19 | 963 | 50.50 (0.30) | 50.61 (0.31) | 0.75 (0.00) |
| ≥ 20 | 578 | 50.43 (0.38) | 48.60 (0.41) | 0.73 (0.01) |
| Main reason for migration^a^ |  |  |  |  |
| Family/partnership reasons | 2040 | 51.97 (0.21)** | 51.81 (0.22)*** | 0.77 (0.00)*** |
| Economic reasons | 1308 | 52.24 (0.26) | 51.67 (0.28) | 0.78 (0.00) |
| Political reasons | 475 | 50.51 (0.44) | 49.49 (0.46) | 0.74 (0.01) |
| Steady relationship before migration |  |  |  |  |
| Yes | 2540 | 52.45 (0.20)*** | 50.70 (0.21)*** | 0.77 (0.00) |
| No | 1584 | 51.08 (0.21) | 52.56 (0.23) | 0.77 (0.00) |
| Connectedness with country of origin |  |  |  |  |
| Very strong | 766 | 51.16 (0.34)* | 51.43 (0.36)* | 0.76 (0.01)* |
| Strong | 1234 | 52.15 (0.27) | 52.25 (0.28) | 0.78 (0.00) |
| In some respects | 1293 | 51.63 (0.26) | 51.31 (0.28) | 0.76 (0.00) |
| Hardly | 560 | 51.69 (0.39) | 51.46 (0.42) | 0.77 (0.01) |
| Not at all | 272 | 53.20 (0.57) | 50.34 (0.61) | 0.78 (0.01) |
| Feeling German |  |  |  |  |
| Entirely | 607 | 53.23 (0.38)** | 51.42 (0.40) | 0.78 (0.01)** |
| Predominantly | 1033 | 51.70 (0.29) | 52.09 (0.31) | 0.77 (0.00) |
| In some respects | 1533 | 51.53 (0.24) | 51.72 (0.25) | 0.77 (0.00) |
| Hardly | 598 | 51.72 (0.38) | 50.88 (0.41) | 0.76 (0.01) |
| Not at all | 347 | 51.08 (0.50) | 50.80 (0.53) | 0.75 (0.01) |
| Disadvantages due to origin |  |  |  |  |
| Often | 411 | 48.80 (0.46)*** | 50.05 (0.50)** | 0.73 (0.01)*** |
| Rarely | 1379 | 51.02 (0.25) | 51.37 (0.27) | 0.76 (0.00) |
| Never | 2335 | 52.81 (0.19) | 51.95 (0.21) | 0.78 (0.00) |
| Oral ability in the German language |  |  |  |  |
| Very good | 1174 | 51.55 (0.27) | 54.13 (0.28)*** | 0.78 (0.00)*** |
| Good | 1365 | 51.79 (0.25) | 51.79 (0.26) | 0.77 (0.00) |
| Not bad | 1136 | 52.08 (0.28) | 50.11 (0.29) | 0.76 (0.00) |
| Fairly bad | 391 | 52.01 (0.47) | 47.89 (0.50) | 0.74 (0.01) |
| Not at all | 57 | 51.04 (1.25) | 47.87 (1.31) | 0.75 (0.02) |

Comparisons of mean MCS, PCS, and SF-6D index scores by migration-related characteristics were analyzed using F tests

MCS: Mental Component Summary; PCS: Physical Component Summary; SE: standard error

* p<0.05, ** p≤0.01, *** p≤0.001

^a^ ‘Other main reason for migration’ is not shown

**Supplementary Table 5.** Multilevel mixed-effects linear regression of the SF-6D index and selected sociodemographic and migration-related characteristics with cluster robust standard errors (years 2014, 2016, 2018, 2020; n = 4124; 9419 observations)

| **Variable** | **Model 3 (dependent variable SF-6D index)** | | |
| --- | --- | --- | --- |
|  | **Coeff.** | **95% CI** | ***p*-value** |
| Gender (Ref. male) |  |  |  |
| Female | −0.00 | −0.01; 0.00 | 0.288 |
| Age, years | *−0.00* | *−0.00; −0.00* | *< 0.001* |
| Marital status (Ref. married/in partnership) |  |  |  |
| Never married/single | −0.00 | −0.01; 0.01 | 0.912 |
| Widowed | −0.00 | −0.02; 0.01 | 0.495 |
| Separated/divorced | −0.00 | −0.01; 0.01 | 0.647 |
| Employment (Ref. unemployed) |  |  |  |
| Employed | *0.04* | *0.03; 0.04* | *< 0.001* |
| School-leaving qualification (Ref. secondary general school)^a^ |  |  |  |
| Secondary school | −0.01 | −0.01; 0.00 | 0.113 |
| Academic secondary school | 0.00 | −0.01; 0.01 | 0.935 |
| No school-leaving qualification | −0.01 | −0.02; 0.01 | 0.392 |
| Country of birth (Ref. Russia) |  |  |  |
| Romania | *0.02* | *0.00; 0.03* | *0.041* |
| Kazakhstan | *0.01* | *0.00; 0.03* | *0.037* |
| Turkey | −0.01 | −0.02; 0.00 | 0.150 |
| Other East Europe^b^ | 0.00 | −0.01; 0.02 | 0.596 |
| Other Europe^c^ | 0.00 | −0.01; 0.01 | 0.868 |
| Other Asia^d^ | −0.01 | −0.00; 0.00 | 0.142 |
| Africa | −0.01 | −0.03; 0.01 | 0.498 |
| America/Oceania | 0.01 | −0.02; 0.03 | 0.567 |
| Religious affiliation (Ref. Christian) |  |  |  |
| Muslim | −0.01 | −0.02; 0.01 | 0.354 |
| Other faith | 0.01 | −0.01; 0.02 | 0.538 |
| Non-denominational | −0.01 | −0.01; 0.00 | 0.167 |
| Time since migration to Germany, years | *−0.00* | *−0.00; −0.00* | *< 0.001* |
| German citizenship (Ref. yes) |  |  |  |
| No | 0.01 | −0.00; 0.01 | 0.192 |
| Main reason for migration (Ref. family/partnership)^e^ |  |  |  |
| Economic reasons | 0.00 | −0.01; 0.01 | 0.439 |
| Political reasons | −0.01 | −0.02; 0.01 | 0.347 |
| Steady relationship before migration (Ref. yes) |  |  |  |
| No | −*0.01* | −*0.02;* −*0.00* | *0.025* |
| Connectedness with country of birth (Ref. very strong) |  |  |  |
| Strong | 0.00 | −0.00; 0.01 | 0.284 |
| In some respects | −0.00 | −0.01; 0.01 | 0.430 |
| Hardly | 0.00 | −0.01; 0.01 | 0.529 |
| Not at all | 0.01 | −0.00; 0.02 | 0.164 |
| Feeling German (Ref. entirely) |  |  |  |
| Predominantly | *−0.01* | *−0.02; −0.00* | *0.013* |
| In some respects | *−0.02* | *−0.03; −0.01* | *< 0.001* |
| Hardly | *−0.02* | *−0.03; −0.01* | *< 0.001* |
| Not at all | *−0.02* | *−0.04; −0.01* | *0.001* |
| Disadvantages due to origin (Ref. often) |  |  |  |
| Rarely | *0.01* | *0.00; 0.03* | *0.019* |
| Never | *0.03* | *0.02; 0.05* | *< 0.001* |
| Oral ability in the German language (Ref. very good) |  |  |  |
| Good | −0.00 | −0.01; 0.01 | 0.565 |
| Not bad | −0.01 | −0.02; 0.00 | 0.082 |
| Fairly bad | *−0.02* | *−0.03; −0.01* | *0.004* |
| Not at all | −0.02 | −0.05; 0.02 | 0.324 |
| Survey year (Ref. 2014) |  |  |  |
| 2016 | 0.01 | −0.00; 0.01 | 0.058 |
| 2018 | 0.00 | −0.01; 0.01 | 0.383 |
| 2020 | *−0.02* | *−0.03; −0.01* | *< 0.001* |
| Constant | *0.85* | *0.82; 0.87* | *< 0.001* |

CI: confidence interval

^a^ ‘Other school-leaving qualification’ is not shown

^b^ Without Russia, Turkey, and Romania

^c^ Without East Europe

^d^ Without Kazakhstan

^e^ ‘Other main reason for migration’ is not shown
